# Temporal trends of physical fitness in northern Italian children (2014–2019): a repeated cross-sectional study

**DOI:** 10.1093/pubmed/fdag020

**Published:** 2026-03-05

**Authors:** Alessandro Gatti, Matteo Giuriato, Matteo Vandoni, Giovanni Messina, Rita Malavolta, Caterina Cavallo, Agnese Pirazzi, Vittoria Carnevale Pellino, Nicola Lovecchio, Stefano Lazzer

**Affiliations:** Laboratory of Adapted Motor Activity (LAMA), Department of Public Health, Experimental Medicine and Forensic Science, University of Pavia, via forlanini 2, Pavia 27100, Italy; National PhD Programme in One Health approaches to infectious diseases and life science research, Department of Public Health, Experimental and Forensic Medicine, University of Pavia, via forlanini 2, Pavia 27100, Italy; Laboratory of Adapted Motor Activity (LAMA), Department of Public Health, Experimental Medicine and Forensic Science, University of Pavia, via forlanini 2, Pavia 27100, Italy; Department of Education and Sport Sciences, Pegaso University, Via Giovanni Porzio, 4, 80143 Napoli, Italy; Laboratory of Adapted Motor Activity (LAMA), Department of Public Health, Experimental Medicine and Forensic Science, University of Pavia, via forlanini 2, Pavia 27100, Italy; Regional School of Sport, Italian Olympic Committee, Via dei Macelli, 5, 34148 Trieste, Italy; School of Sport Sciences, University of Udine, p.le D. Simonetti, 2, 33013 Gemona del Friuli, Udine, Italy; Regional Italian Olympic Committee, Via dei Macelli, 5, 34148 Trieste, Italy; Laboratory of Adapted Motor Activity (LAMA), Department of Public Health, Experimental Medicine and Forensic Science, University of Pavia, via forlanini 2, Pavia 27100, Italy; Department of Sport, LUNEX University of Applied Sciences, 50, Avenue du Parc des Sports, Differdange 4671, Luxembourg, Luxembourg; Laboratory of Adapted Motor Activity (LAMA), Department of Public Health, Experimental Medicine and Forensic Science, University of Pavia, via forlanini 2, Pavia 27100, Italy; Laboratory of Adapted Motor Activity (LAMA), Department of Public Health, Experimental Medicine and Forensic Science, University of Pavia, via forlanini 2, Pavia 27100, Italy; Department of Education and Sport Sciences, Pegaso University, Via Giovanni Porzio, 4, 80143 Napoli, Italy; Department of Human and Social Science, University of Bergamo, via Salvecchio 19, Bergamo 24127, Italy; School of Sport Sciences, University of Udine, p.le D. Simonetti, 2, 33013 Gemona del Friuli, Udine, Italy; Department of Medicine, University of Udine, via Palladio 8, 33100 Udine, Italy

**Keywords:** exercise, testing, fitness, trends, youth

## Abstract

**Background:**

Physical fitness (PF) is a crucial indicator of long-term health in children, influencing risks for cardiovascular disease, obesity, and overall mortality. Despite its significance, Italy lacks a national surveillance system able to track PF trends in children, hindering efforts to combat rising obesity rates. This study aims to evaluate temporal trends in PF through a possible surveillance system in elementary school children.

**Methods:**

A repeated cross-sectional study was performed and consisted of assessing five PF domains: balance, upper and lower limb strength, cardiorespiratory fitness (CRF), and, speed-agility, along with BMI z-scores. PF trends were analyzed by age and sex, with logistic regression assessing the link between PF and obesity risk. Effects sizes (ES, Cohen’s d) were computed to describe the trend’s magnitude.

**Results:**

CRF improved across all ages, especially in 10–11-year-olds (ES > 1.00). Younger children (6–9 years) showed gains in speed-agility, upper and lower limb strength, but these plateaued in older groups. We observed a decline in balance in 10-year-old boys.

**Conclusions:**

Overall, PF levels increased over time, with the most notable improvements observed in CRF. Implementing a nationwide PF surveillance system would facilitate continuous tracking of fitness trends, enabling policymakers to identify declines and develop targeted interventions.

## Introduction

Physical fitness (PF) involves different domains, including health-related and skill-related components, and refers to the ability to perform physical activity and movements efficiently and effectively. Health-related PF includes several key components such as cardiorespiratory fitness (CRF), muscular strength (MS), and flexibility, which are closely linked to all-cause mortality and associated with various health outcomes.^1^^,^^2^ For instance, both CRF and MS are associated with cardiovascular health and lipid metabolism.^1^^,^^2^

CRF levels are better predictors of all-cause mortality than conventional risk factors such as smoking, hypertension, high cholesterol, and diabetes.^3^ Skill-related PF instead refers to a wide range of domains (i.e. muscular power, speed-agility, balance) implied in sports or activities and related to motor competencies.^4^ Although this categorization appears to divide PF into distinct domains, in reality, skill-related factors influence health-related performance and vice versa.^5^ Although overall PF is a significant determinant of health, it is frequently evaluated exclusively within the context of the health-related domain, with the skill-related domain receiving comparatively limited research attention. Only a few studies with representative samples were found to evaluate skill-related^6^^,^^7^ and most of the studies are focused especially on CRF.^6^^,^^8^

In addition, due to the rising obesity rates in children,^9^ evaluating the PF temporal trends in children and adolescents has become a topic of interest for many researchers in past decades. Analyzing temporal trends in youth can reveal the impact of social change and population-based interventions, presenting insights into the future health of young generations.^6^ Moreover, monitoring PF should be considered crucial since it could help monitor the spread of obesity and predict the risk for future health problems.^10^ Due to the relevance of PF for health,^5^ many nations^6^^,^^8^ started promoting national surveillance systems to track PF levels of the youth. For example, in 2008, Japan implemented the National Survey of Physical Fitness, Athletic Performance, and Exercise Habits (hereafter called the JP Fit).^8^ Even if this surveillance system was limited to 5^th^ and 8^th^ grade students, it allowed testing 97% of the students from 2013 at the school level. Moreover, in Slovenia, a national monitoring system to evaluate changes in PF of children and adolescents was developed in 1969, and partially thanks to this system, Slovenian schoolchildren and adolescents are among the most active in the world.^11^ Unfortunately while there is a surveillance system to assess and track physical activity levels in the Italian population,^12^ a monitoring system to evaluate changes in PF levels, even if critical, has not been implemented. Since reductions in PF levels were frequently observed,^13^^,^^14^ this gap is concerning and could be attributed to a decline in physical activity levels and changes in body composition.^9^^,^^15^ Considering the reported falling trends,^16^ some researchers foresee the potential negative effect of poor PF for significant public health issues.^15^

Given these downward trends, some Italian regions tried to implement new monitoring assessments for PF levels. In line with these efforts, we introduced a PF surveillance system to monitor the PF and anthropometric characteristics of elementary schoolchildren.^17^ For this reason, the primary aim of this study is to determine the temporal trends of the PF levels of 6- to 11-year-old schoolchildren between 2014 and 2019.

## Methods

### Study protocol

The data described in this study follows the STrengthening the Reporting of OBservational studies in Epidemiology (STROBE) for reporting cross-sectional results were obtained from the surveillance system started in a northern region of Italy called ‘MOVIMENTO in 3S: promozione della SALUTE nelle SCUOLE attraverso lo SPORT’ (MOVIMENTO in 3S: promoting Health in Schools through Sport). Through the collection of PF tests and anthropometric data, the project aimed at monitoring how the PF changed over time. The University of Udine Ethics Committee on Human Research for Medical Sciences approved the experimental protocol (N. 74/2010), and the study was conducted following the guidelines provided by the Declaration of Helsinki.^18^ Before giving consent to participate in the study, purpose, and objectives were thoroughly explained to each child and their parents. Then, verbal consent was obtained from the children, and their parents or legal tutor provided written informed consent.

### Participants

**Figure 1:**
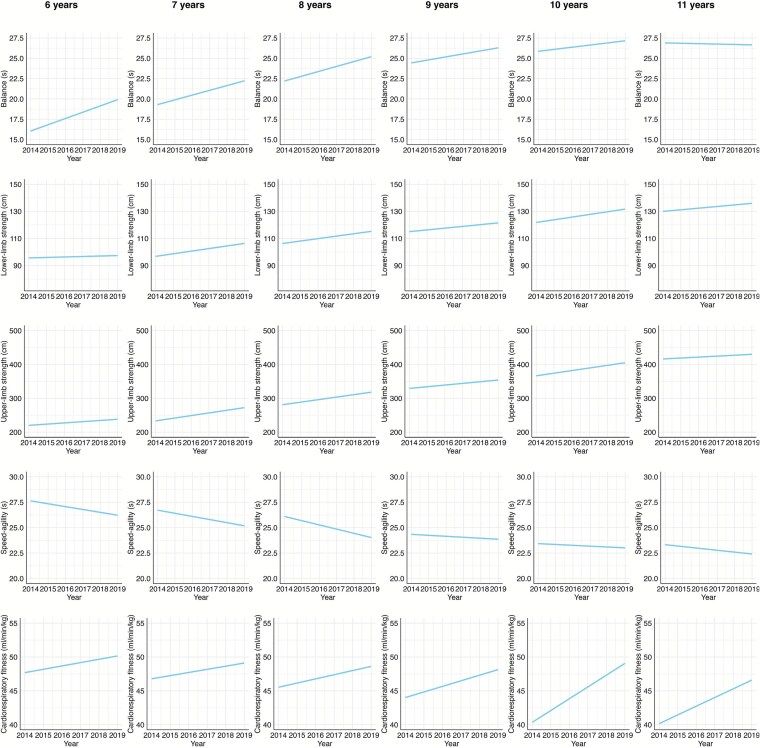
Temporal trends in different PF domains for 6 to 11 years old girls (from left to right) from 2014 to 2019. The values in the graph are estimates obtained in the regression, adjusted for BMI z-score and peak height velocity. The fitness components measured included balance (single leg standing, 1, 2, 3, 4, 5, 6) in seconds (s), lower-limb strength (standing broad jump—SBJ, 7, 8, 9, 10, 11, 12) in centimeters (cm), cardiorespiratory fitness (multistage fitness test, 13, 14, 15, 16, 17, 18) in milliliters per minute per kilogram (ml/min/kg), upper-limb strength (medicine ball throw—MBT, 19, 20, 21, 22, 23, 24) in centimeters (cm), and speed-agility (10x5m shuttle run, 25, 26, 27, 28, 29, 30) in seconds (s).

We recruited a total of 150 545 children (aged from 6 to 11 years old; girls 72 200 (48.0%) from 2014 to 2019 (see Fig. 1) and they were considered eligible if they fulfilled the following criteria: attending elementary school, participating in the regular school physical education program, being in good health, and not being involved in other studies. Exclusion criteria included orthopedic injuries that occurred in the past six months, any condition preventing participation in physical education classes, and any medical condition that restricted the ability to engage in exercise. All measurements were conducted during school hours at the school sports facilities, assessing both anthropometric measurements and physical fitness parameters. All the schools participating in the project were contacted via email by the University of Udine, and they were all located in urban areas. In order to reduce the risk of bias in performing the evaluations and collecting the data, all the evaluators were carefully instructed by the study personnel on how correctly perform and collect the PF tests’ data. Fig. S1 reports the study design and the timeline of the study.

### Anthropometric characteristics

The surveillance system collected data annually at the school level, from mid-September to mid-October. Although some children may have been assessed in more than one year, individual longitudinal follow-up was not considered. Therefore, each annual assessment was treated as an independent observation corresponding to a child-year. Measurements of body mass and height were conducted following the standards set by the International Society for the Advancement of Kinanthropometry.^19^ During these assessments, participants were barefoot and dressed in lightweight exercise clothing (shorts and T-shirt). Body weight was measured with a balance scale (Seca 864, Seca GmbH and Co., Hamburg, Germany), accurate to 0.1 kg. Height was recorded using a stadiometer (Seca 216, Seca GmbH and Co., Hamburg, Germany), with a precision of 1 cm, while participants stood upright with their heads aligned in the Frankfort plane. The collected data were used to calculate the BMIz-score according to the World Health Organization Growth (WHO) chart and equation.^20^ Children were classified as with obesity if they had a BMI z-score higher than > 2 SD, as classified by the WHO. Additionally, using a modified equation by Kozieł et al.,^21^ height and age were used to predict Maturity offset:

For girls:

Maturity offset (years) = −7.709133 + (0.0042232 × (age × height)).

For boys:

Maturity offset (years) = −7.999994 + (0.0036124 × (age × height)).

The difference between the chronological age at prediction and the maturity offset provided an estimate of the age at peak height velocity (PHV). This formula was chosen because it allows for an accurate estimation of PHV using only simple anthropometric measures, comparable to the accuracy of more complex equations.^21^

### Physical fitness test battery

#### Protocol assessments

The evaluation process included a series of comprehensive PF assessments commonly performed in educational settings. The practical tests used in this evaluation are reliable and valid tools for measuring the PF of young people and adolescents. Furthermore, these assessments are standardized, affordable in terms of necessary equipment, and simple to conduct.^22^ To guarantee that all sports scientists executed the tests systematically, a seminar was held to thoroughly explain the procedures for conducting and documenting the evaluations. Additionally, the sports scientists were instructed to administer the PF tests in a consistent order to reduce potential inter-rater reliability issues among the evaluators.

#### Static balance

Children performed the standing balance test to assess their static balance level.^23^ They balanced on their dominant leg, keeping the other leg flexed at the knee. Each child had 1 minute to practice before the actual test, during which they stood on their dominant leg. At the signal, the child lifted the dominant heel from the floor, trying to maintain balance for up to 30 seconds. The trial concluded if the child moved their hands from the hips, shifted the supporting foot, or if the non-supporting foot moved from its position. A stopwatch recorded the total time in seconds as the heel was raised from the floor, up to a maximum of 30 seconds.

#### Upper body strength

The assessment of upper body explosive strength involved a backward throw of a 1 or 2 kg medicine ball (1 kg for children in age classes I and II, and 2 kg for those in classes III, IV, and V). Participants were instructed to throw the ball as far as possible using both hands, sitting on the ground with their legs apart and their back leaning against the wall.^22^^,^^24^ Each child performed the test twice, and the longest throw, measured in centimeters, was recorded.

#### Lower limb strength

Lower limb explosive strength was assessed using a standing broad jump test.^22^ In this test, each child started from a standing position and jumped as far as possible. To execute the jump, the children were instructed to bend their knees, extend their arms forward parallel to the ground, and then swing their arms back and forth while pushing off forcefully to achieve maximum distance. They were instructed to land with their feet together and maintain their balance. Each child performed the jump twice, with the greatest distance recorded in centimeters.

#### Cardiorespiratory fitness

The 20-meter shuttle run test, or ‘Multistage fitness test,’ is one of the most used field assessments for evaluating CRF.^25^ This test is straightforward, easy to administer, and not time-consuming. It requires minimal equipment, and it allows testing many individuals at once. The test involves running back and forth between two 20-meter lines in synchronization with pre-recorded audio cues. It begins at a speed of 8.5 km/h, increasing by 0.5 km/h every minute.^26^ The test concludes when a participant fails to reach the lines in sync with the audio cues twice in a row.

Then after the test was concluded the VO_2_ max was estimated through the following equation^26^:

VO_2_ max = 31.025 + 3.238 * Speed (km/h)—3.248 * Age + 0.1536 * Age * Speed (km/h).

#### Speed-agility

Speed-agility was evaluated using a 10 × 5-meter shuttle run test.^27^ For this test, two parallel lines were marked on the floor 5 meters apart. The participant was instructed to run as quickly as possible from the starting line to the second line and back, ensuring at least one foot crossed each line on every lap. This process was repeated five times, resulting in a total distance of 50 meters (10 × 5 meters). The timing ended when the participant crossed the finish line with one foot. The duration was measured with a standard stopwatch.

### Statistical analysis

All data were manually entered into a spreadsheet and checked for transcription errors. Our analysis was stratified into groups based on test, sex and age (e.g. 11-year-old boys undergoing the standing broad jump test). As underlined by recent literature,^28^^,^^29^ maturity and BMIz-score significantly impact PF test performance, therefore, analyses were adjusted for anthropometric characteristics and maturity offset. BMIz-score-maturity-adjusted temporal trends in PF tests were determined using an analysis of covariance (ANCOVA, based on a linear regression model) or a weighted least squares regression for each test-sex-age group, with corresponding 95% confidence interval (CI^30^). Model selection was informed by the Breusch-Pagan test for heteroskedasticity. These trends were then expressed per 5-years in absolute terms (regression coefficient), percent trends (regression coefficient as a percentage of the mean), and standardized Cohen’s ES (regression coefficient divided by the SD, with ES thresholds of 0.2, 0.5, and 0.8 for small, moderate, and large, respectively, and ES < 0.2 considered negligible). Population-weighted mean trends were calculated for boys, girls, and the total sample using post-stratification weights based on ISTAT sex- and age-specific population estimates for Italy. As a sensitivity analysis, generalized linear mixed models (GLMM) were fitted to account for potential within-child clustering due to repeated measurements in a subset of participants, including fixed effects (BMI z-score and maturity offset) and random intercepts for individuals. Annual trends were summarized using unstandardized year coefficients with 95% confidence intervals (Supplementary Figs S2–S11; Tables S1–S5). To further explain the model’s explanatory power, we included the R-squared (R^2^) values, which indicate the proportion of variance explained by the model. Higher R^2^ values indicate a better model fit. Additionally, we created graphical representations based on the model’s estimates to visually illustrate the trends and relationships over time. For these analyses, a p-value of < 0.05 was used to determine statistical significance. All analyses were conducted using R software, version 4.4 (R Foundation for Statistical Computing), the ‘zscorer’ package was used to compute the BMIz-score and the ‘lme4’ package to perform the GLMM.

## Results

The descriptive characteristics of the total sample, divided by sex, are presented in Table 1.

**Table 1:** Descriptive characteristics of the study sample.

	All (n = 150,545)	Boys (n = 78,345)	Girls (n = 72,200)
Age (years)	8.48 (8.47–8.49)	8.50 (8.48–8.51)	8.46 (8.45–8.47)
Height (m)	1.31 (1.31–1.31)	1.32 (1.31–1.32)	1.31 (1.31–1.31)
Weight (kg)	30.51 (30.46–30.55)	30.78 (30.72–30.84)	30.21 (30.15–30.27)
Body mass index z-score	0.56 (0.56–0.57)	0.54 (0.53–0.55)	0.59 (0.58–0.60)
Peak Height Velocity	−3.48 (−3.49—−3.47)	−3.93 (−3.93—−3.92)	−3.00 (−3.01—−2.99)
*Physical fitness tests*			
Balance (s)	22.31 (22.27–22.36)	21.01 (20.95–21.07)	23.72 (23.66–23.78)
Lower-limb explosive strength (cm)	117.37 (117.25–117.49)	120.94 (120.77–121.11)	113.51 (113.35–113.68)
Upper-limb explosive strength (cm)	332.84 (332.38–333.31)	347.68 (347.01–348.35)	316.88 (316.25–317.51)
Speed-agility (s)	24.35 (24.33–24.38)	24.00 (23.97–24.03)	24.74 (24.71–24.78)
Maximal-speed CRF (km/h)	9.74 (9.73–9.74)	9.88 (9.87–9.89)	9.58 (9.57–9.59)
VO_2_ Max CRF (ml/kg/min)	48.15 (48.12–48.18)	48.78 (48.74–48.82)	47.46 (47.42–47.50)

Data are presented as mean (95% CI). CRF = Cardiorespiratory fitness.

The adjusted temporal trends for PF performance are detailed in Table 2.

**Table 2:** Temporal trends in means for BMI-maturity-adjusted physical fitness among northern Italian children aged 6–11 years between 2014–2019 for boys and girls.

				Trends in means (95% CI)		
	Sex	Age	N	Absolute	Percent	Standardized ES
BMIzscore-maturity-adjusted balance test (s)	Boys	6	5630	2.14 (2.02, 2.27)	14.39 (14.06, 14.72)	0.22 (0.18, 0.26)
	Boys	7	17 771	0.72 (0.54, 0.89)	4.08 (3.66, 4.50)	0.08 (0.02, 0.14)
	Boys	8	17 241	1.11 (0.89, 1.32)	5.81 (5.32, 6.30)	0.13 (0.05, 0.20)
	Boys	9	14 424	0.71 (0.57, 0.85)	3.12 (2.82, 3.42)	0.09 (0.04, 0.14)
	Boys	10	11 376	−4.85 (−5.03, −4.67)	−19.89 (−20.26, −19.52)	−0.67 (−0.75, −0.60)
	Boys	11	7904	−1.19 (−1.25, −1.14)	−4.75 (−4.86, −4.64)	−0.19 (−0.21, −0.16)
	Girls	6	5168	3.72 (3.55, 3.89)	19.89 (19.49, 20.28)	0.37 (0.32, 0.43)
	Girls	7	16 929	2.54 (2.20, 2.87)	12.13 (11.40, 12.86)	0.30 (0.18, 0.41)
	Girls	8	16 177	2.74 (2.41, 3.08)	11.52 (10.83, 12.22)	0.36 (0.24, 0.49)
	Girls	9	13 262	1.79 (1.57, 2.02)	7.07 (6.63, 7.52)	0.27 (0.18, 0.36)
	Girls	10	10 622	1.10 (1.01, 1.19)	4.16 (3.99, 4.34)	0.19 (0.15, 0.23)
	Girls	11	6939	0.31 (0.28, 0.34)	1.16 (1.10, 1.22)	0.06 (0.05, 0.07)
BMIzscore-maturity-adjusted SBJ test (cm)	Boys	6	5597	−0.52 (−0.58, −0.46)	−0.51 (−0.57, −0.44)	−0.03 (−0.04, −0.01)
	Boys	7	17 774	7.15 (6.59, 7.70)	8.53 (7.93, 9.14)	0.35 (0.23, 0.48)
	Boys	8	17 305	7.69 (7.12, 8.25)	6.47 (5.95, 6.99)	0.37 (0.25, 0.50)
	Boys	9	14 419	5.52 (5.12, 5.92)	4.40 (4.04, 4.75)	0.26 (0.17, 0.35)
	Boys	10	11 455	4.82 (4.63, 5.00)	3.60 (3.44, 3.76)	0.22 (0.18, 0.26)
	Boys	11	7833	2.68 (2.59, 2.76)	1.91 (1.84, 1.98)	0.12 (0.10, 0.14)
	Girls	6	5168	0.71 (0.63, 0.78)	0.73 (0.66, 0.81)	0.04 (0.02, 0.05)
	Girls	7	16 945	7.85 (7.27, 8.44)	7.69 (7.11, 8.28)	0.43 (0.29, 0.57)
	Girls	8	14 419	7.59 (7.03, 8.14)	6.84 (6.31, 7.36)	0.39 (0.26, 0.52)
	Girls	9	13 209	5.22 (4.85, 5.60)	4.43 (4.08, 4.78)	0.26 (0.17, 0.35)
	Girls	10	10 658	7.56 (7.33, 7.79)	6.00 (5.79, 6.21)	0.36 (0.30, 0.41)
	Girls	11	6989	4.88 (4.76, 5.01)	3.69 (3.58, 3.80)	0.23 (0.20, 0.25)
BMIzscore-PHV-adjusted medicine ball throw test (cm)	Boys	6	5541	13.41 (13.09, 13.73)	5.31 (5.11, 5.51)	0.24 (0.20, 0.28)
	Boys	7	17 492	26.68 (25.61, 27.74)	9.64 (9.00, 10.28)	0.43 (0.29, 0.56)
	Boys	8	17 024	20.12 (19.21, 21.02)	6.11 (5.61, 6.61)	0.30 (0.19, 0.41)
	Boys	9	14 183	12.04 (11.46, 12.63)	3.21 (2.91, 3.51)	0.16 (0.09, 0.23)
	Boys	10	11 196	6.49 (6.28, 6.71)	1.54 (1.43, 1.64)	0.09 (0.06, 0.11)
	Boys	11	7721	0.01 (0.01, 0.02)	0.00 (0.00, 0.01)	0.00 (0.00, 0.00)
	Girls	6	5138	11.86 (11.55, 12.16)	5.10 (4.90, 5.30)	0.24 (0.20, 0.29)
	Girls	7	16 687	25.08 (24.04, 26.12)	9.84 (9.19, 10.50)	0.46 (0.31, 0.60)
	Girls	8	14 183	21.78 (20.84, 22.71)	7.24 (6.70, 7.78)	0.37 (0.24, 0.49)
	Girls	9	13 046	11.63 (11.07, 12.18)	3.41 (3.10, 3.71)	0.18 (0.11, 0.25)
	Girls	10	10 471	17.13 (16.78, 17.48)	4.47 (4.29, 4.65)	0.24 (0.20, 0.29)
	Girls	11	6920	3.21 (3.10, 3.31)	0.76 (0.71, 0.81)	0.04 (0.03, 0.06)
BMIzscore-maturity-adjusted VO_2_ max (Leger test, ml /min/kg)	Boys	6	1661	2.07 (1.96, 2.17)	4.10 (3.96, 4.25)	0.42 (0.37, 0.47)
	Boys	7	3088	2.27 (2.13, 2.42)	4.60 (4.40, 4.80)	0.44 (0.38, 0.50)
	Boys	8	3670	2.37 (2.20, 2.54)	4.81 (4.57, 5.06)	0.42 (0.35, 0.49)
	Boys	9	2882	3.54 (3.36, 3.73)	7.31 (7.04, 7.58)	0.63 (0.55, 0.71)
	Boys	10	1962	6.70 (6.55, 6.86)	14.02 (13.80, 14.25)	1.07 (1.00, 1.13)
	Boys	11	950	6.35 (6.25, 6.45)	13.88 (13.73, 14.02)	0.97 (0.93, 1.01)
	Girls	6	1255	2.35 (2.24, 2.45)	4.74 (4.59, 4.88)	0.63 (0.58, 0.68)
	Girls	7	2886	2.26 (2.12, 2.41)	4.66 (4.45, 4.86)	0.49 (0.42, 0.55)
	Girls	8	2882	2.91 (2.72, 3.10)	6.09 (5.82, 6.37)	0.61 (0.52, 0.69)
	Girls	9	2695	3.90 (3.71, 4.10)	8.35 (8.46, 8.81)	0.76 (0.68, 0.85)
	Girls	10	1729	8.64 (8.46, 8.81)	18.80 (18.54, 19.06)	1.56 (1.49, 1.64)
	Girls	11	787	6.33 (6.22, 6.44)	14.29 (14.13, 14.45)	1.14 (1.10, 1.19)
BMIzscore-maturity-adjusted shuttle run test (s)	Boys	6	5052	−1.16 (−1.30, −1.02)	−4.49 (−4.77, −4.20)	−0.26 (−0.33, −0.19)
	Boys	7	10 751	−1.70 (−1.95, −1.44)	−6.83 (−7.33, −6.32)	−0.38 (−0.50, −0.26)
	Boys	8	10 532	−1.43 (−1.65, −1.20)	−5.99 (−6.46, −5.52)	−0.33 (−0.44, −0.22)
	Boys	9	7313	−1.31 (−1.49, −1.13)	−5.62 (−5.99, −5.24)	−0.28 (−0.36, −0.20)
	Boys	10	5111	−0.05 (−0.07, −0.03)	−0.23 (−0.27, −0.19)	−0.01 (−0.02, 0.00)
	Boys	11	2415	−0.14 (−0.16, −0.12)	−0.62 (−0.66, −0.58)	−0.04 (−0.05, −0.03)
	Girls	6	4637	−1.31 (−1.46, −1.15)	−4.90 (−5.20, −4.60)	−0.29 (−0.37, −0.22)
	Girls	7	9917	−1.32 (−1.54, −1.10)	−5.18 (−5.61, −4.74)	−0.31 (−0.42, −0.21)
	Girls	8	7313	−1.98 (−2.24, −1.72)	−8.06 (−8.59, −7.53)	−0.46 (−0.58, −0.33)
	Girls	9	6824	−0.39 (−0.49, −0.29)	−1.63 (−1.82, −1.43)	−0.09 (−0.14, −0.04)
	Girls	10	4661	−0.19 (−0.23, 0.15)	−0.82 (−0.89, 0.74)	−0.05 (−0.07, −0.03)
	Girls	11	1995	−0.83 (−0.88, −0.78)	−3.66 (−3.77, −3.56)	−0.24(−0.27, −0.21)
Maturity-adjusted BMI z-score	Boys	6	5581	−0.13 (−0.17, −0.10)	−23.32 (−23.75, −22.89)	−0.12 (−0.15, −0.09)
	Boys	7	17 855	0.03 (0.01, 0.07)	5.69 (5.19, 6.18)	0.03 (−0.01, 0.06)
	Boys	8	17 307	0.05 (0.00, 0.09)	9.01 (8.40, 9.62)	0.05 (0.00, 0.09)
	Boys	9	14 545	0.10 (0.05, 0.16)	17.73 (17.03, 18.44)	0.10 (0.04, 0.15)
	Boys	10	11 609	0.05 (0.03, 0.06)	8.42 (8.18, 8.66)	0.04 (0.03, 0.06)
	Boys	11	7991	0.01 (0.00, 0.01)	1.93 (1.86, 2.00)	0.01 (0.00, 0.01)
	Girls	6	5137	0.05 (0.03, 0.07)	7.88 (7.63, 8.14)	0.03 (0.02, 0.05)
	Girls	7	16 936	0.05 (0.00, 0.09)	8.35 (7.75, 8.95)	0.03 (0.00, 0.07)
	Girls	8	16 140	0.01 (−0.01, 0.03)	1.81 (1.54, 2.08)	0.01 (−0.01, 0.03)
	Girls	9	13 307	0.08 (0.04, 0.13)	12.44 (11.86, 13.02)	0.06 (0.02, 0.10)
	Girls	10	10 803	0.12 (0.09, 0.15)	19.39 (19.02, 19.76)	0.09 (0.06, 0.12)
	Girls	11	7084	0.06 (0.05, 0.08)	10.95 (10.76, 11.75)	0.05 (0.04, 0.06)

Notes: Positive trends indicated increases in performance and negative trends indicated declines in performance. Only for speed-agility negative trends indicated increases in performance and positive trends indicated declines in performance.

**Figure 2:**
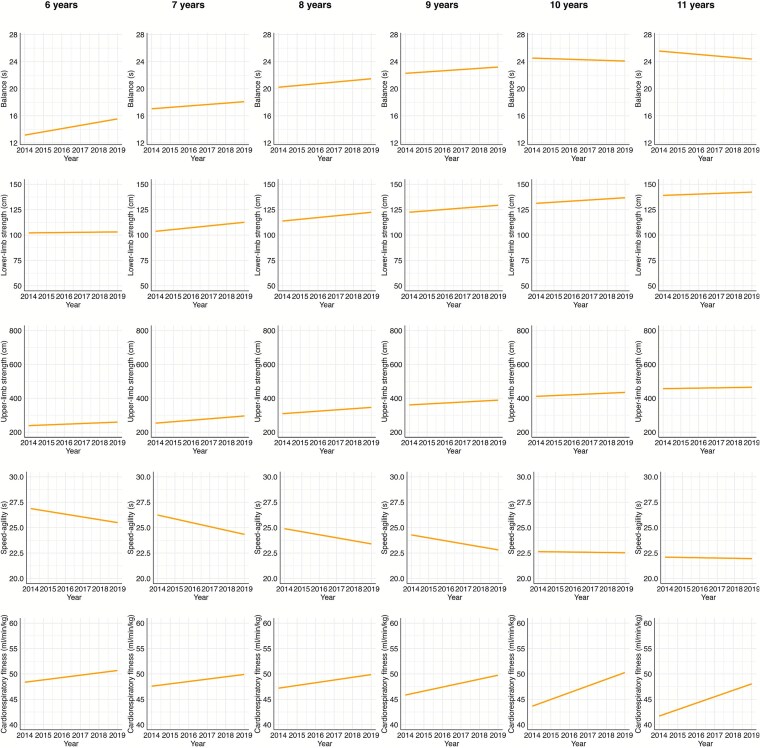
Temporal trends in different PF domains for 6 to 11 years old boys (from left to right) from 2014 to 2019. The values in the graph are estimates obtained in the regression, adjusted for BMI z-score and peak height velocity. The fitness components measured included balance (single leg standing, 1, 2, 3, 4, 5, 6) in seconds (s), lower-limb strength (standing broad jump—SBJ, 7, 8, 9, 10, 11, 12) in centimeters (cm), cardiorespiratory fitness (multistage fitness test, 13, 14, 15, 16, 17, 18) in milliliters per minute per kilogram (ml/min/kg), upper-limb strength (medicine ball throw—MBT, 19, 20, 21, 22, 23, 24) in centimeters (cm), and speed-agility (10x5m shuttle run, 25, 26, 27, 28, 29, 30) in seconds (s).

These trends varied based on the children’s ages. CRF was the only domain that consistently improved across all age groups. For children aged 6 to 9, there was a notable improvement in speed-agility, as well as upper and lower limb MS for both boys and girls (Effect Size, ES, > 0.20). However, for those aged 10 and 11, performance in these areas remained stable (−0.20 < ES < 0.20). Notably, balance performance for 10- and 11-year-olds declined from 2014 to 2019 (ES < −0.20). To facilitate the visualization of these trends, we provided also a graphical representation of the regression estimates grouped by sex (Fig. 1 girls, and Fig. 2 boys).

Specifically, estimated VO₂ max for boys increased by 2.07–3.54 ml ·kg^−1^·min^−1^ between ages 6 and 9 (ES = 0.42–0.63), with markedly larger gains observed at ages 10 and 11 (6.70 [95% CI: 6.55, 6.86] and 6.35 [6.25, 6.45] ml ·kg^−1^·min^−1^, corresponding to ES = 1.07 and 0.97, respectively). Similar patterns were observed in girls, with VO₂ max increases ranging from 2.35 to 3.90 ml ·kg^−1^·min^−1^ at ages 6–9 (ES = 0.49–0.76) and peaking at ages 10–11 (8.64 [8.46, 8.81] and 6.33 [6.22, 6.44] ml ·kg^−1^·min^−1^; ES = 1.56 and 1.14, respectively).

Lower-limb muscular strength, assessed by the SBJ, had small improvement primarily in younger children. Among boys aged 7–9 years, jump distance increased by 5.52–7.69 cm (ES = 0.26–0.37), while girls of the same age showed comparable gains (5.22–7.85 cm; ES = 0.26–0.43). In contrast, changes in older children were smaller, with effect sizes approaching negligible magnitude at age 11 (boys: 2.68 cm, ES = 0.12; girls: 4.88 cm, ES = 0.23).

Upper-limb muscular strength, measured by the medicine ball throw, exhibited moderate improvements in younger age groups. In boys aged 6–8 years, throwing distance increased by 13.41–26.68 cm (ES = 0.24–0.43), whereas minimal or negligible changes were observed at age 11 (0.01 cm; ES = 0.00). A similar age-related attenuation was observed in girls, with moderate improvements at ages 6–8 (11.86–25.08 cm; ES = 0.24–0.46) and small effects thereafter. Speed-agility performance improved mainly in children aged 6–9 years. Shuttle run time decreased by 1.16–1.70 s in boys (ES = −0.26 to −0.38) and by 1.31–1.98 s in girls (ES = −0.29 to −0.46), indicating faster performance. In contrast, changes in older children were minimal, with effect sizes close to zero at ages 10–11. Balance performance showed divergent age-specific trends. While younger children exhibited small improvements (e.g. boys aged 6: +2.14 s, ES = 0.22; girls aged 6: +3.72 s, ES = 0.37), older children experienced declines. In boys aged 10 years, balance time decreased by 4.85 s (95% CI: −5.03, −4.67; ES = −0.67), with a smaller decline at age 11 (−1.19 s; ES = −0.19). Girls showed more modest reductions or stabilization at older ages (ES ≤ |0.20|). Concurrently, maturity-adjusted BMI z-scores remained stable over time across most age groups in both sexes, with absolute changes ranging from 0.01 to 0.12 z-score units and consistently small effect sizes (ES < 0.20, Fig. S12).

## Discussion

### Main findings of the study

Based on the findings of the ‘Movement in 3S’ study, the present research aimed to assess the temporal trends of PF on elementary school children in Northern Italy. The study focused on evaluating the PF levels and anthropometric characteristics of children aged 6 to 11. The results revealed that while CRF consistently improved across all age groups, notable differences were observed in other fitness domains. For younger children, aged 6 to 9, there was significant improvement in speed-agility and both upper and lower limb strength for both boys and girls. However, the performance in these areas remained stable for children aged 10 and 11. Additionally, a decline in balance performance was noted for older children. These findings highlight the critical role of age and fitness components in designing effective interventions aimed at improving PF. Conventional standardized effect size thresholds guided the interpretation of the observed trends, with values of at least 0.20 considered indicative of non-negligible changes. In the present study, this threshold corresponded to interpretable differences in raw performance. For instance, effect sizes greater than 0.80 in CRF were associated with increases of 6–9 ml ·kg^−1^·min^−1^ in estimated VO₂ max among 10–11-year-old children. Meanwhile, small-to-moderate effects (ES from 0.22 to 0.43) in lower-limb power were associated with gains of ~5–8 cm in standing broad jump performance in children aged 7–10 years. Conversely, effect sizes below 0.20 corresponded to minimal absolute changes (e.g. ≤ 2 cm in jump distance or ≤ 0.5 s in balance time), supporting their classification as negligible in this population. The divergence observed between the substantial improvements in CRF and the stabilization or decline of neuromuscular components in older children indicates exposure to activity stimuli specific to each domain. School-based physical education programs often prioritize aerobic and endurance-based activities, which effectively improve CRF but offer limited opportunities for balance- and strength-oriented tasks.^31^ Additionally, the modest but consistent increases in BMI z-scores observed across several age groups may have counteracted some of the gains in performance in weight-bearing and coordination-dependent tasks. This may contribute to the age-related reduction in improvements in balance and muscular strength.

### What is already known on this topic

Over the past decade, CRF has significantly improved across all child age groups (6–11 years), which contrasts with recent studies. For instance, Li et al.^32^ reported that from 1985 to 2019, the CRF of Chinese adolescents declined sharply. These negative trends were also observed in Hong Kong and Croatia, and similarly documented in the Italian population from 1984 to 2010.^33^^,^^34^ Despite this, only continuous running tests were used in these studies to assess CRF, while we employed an incremental running test. As in our study, the JP Fit Survey for Youth^8^ examined aerobic capacity using the 20-meter shuttle run test in Japan. According to the survey’s findings, participants’ performance in the 20-meter shuttle run test remained relatively stable from 2013 to 2019, indicating consistent fitness levels among adolescents during this period. Moreover, the results from the SLOfit showed how from 2003 to 2013 the CRF levels of 10–13-year-old children improved independently of age and sex.^6^ This improvement in CRF could potentially reflect a reduction in the risk of cardiovascular and metabolic diseases in children,^3^ emphasizing the relevance of monitoring PF trends over time.

Our results outlined upward trends in balance, speed-agility, and lower and upper limb strength among children aged 6 to 9 years, whereas the performance of children aged 10 to 11 years remained stable across these domains, except for a decline in balance observed only in 10-year-old boys. For both balance and speed agility our results are in accordance with the results obtained by the surveillance system implemented in Japan^8^ but are in contrast with what was found by the SLOfit,^6^ which reported a descending trend in both speed and balance for 6–10 children. These results reflect also the contrasting literature about these two tests. For instance, while some studies report a downward trend,^35^^,^^36^ other reports a stagnation^37^ or even a rise^7^^,^^38^ in the performance of these PF domains over the years. Despite this, the stabilization in balance and speed-agility can be attributed to the fact that coordination tends to stabilize between the ages of 10 and 13.^39^

The trends in MS performance contrast with most of the existing literature. For example, studies in UK, Spain, and Lithuania, as well as a large meta-analysis on European children, reveal a downward trend in MS test performance.^7^^,^^38^^,^^40^ Considering that even the JP Fit Survey for Youth^8^ reported a stable trend in MS performance before the COVID-19 outbreak. Since MS levels in childhood often reflect those in adulthood and are closely linked to long-term health outcomes,^41^ there is a need of further investigation on MS for reducing future health risks and enhancing overall well-being.

### What this study adds

This study contributes valuable insights into the trends of physical fitness among Italian elementary school children by utilizing a repeated cross-sectional design across five annual assessments. With a large sample size of 150 545 participants from a single region, the findings offer a robust foundation for understanding PF trends and potentially applying these observations nationwide. Unlike previous studies, this research adjusted for critical confounders such as weight and maturity status, enhancing the reliability of the results. By examining multiple fitness components, including CRF, muscular strength, speed-agility, and balance, the study provides a comprehensive overview rather than focusing solely on aerobic capacity. Importantly, the study underscores the relevance of ongoing fitness surveillance in childhood, as PF during these years is closely linked to physical, cognitive, and mental health outcomes later in life.^10^ Overall, these findings underscore the relevance of structured PF surveillance systems that can capture age- and domain-specific trends, rather than relying solely on CRF. The coexistence of substantial improvements in aerobic capacity alongside stagnation or decline in balance and muscular strength suggests that current physical activity strategies may be insufficient for targeting neuromuscular development, especially in older children. From a health policy perspective, integrating age-appropriate strength and motor competence activities into school-based physical education curricula, alongside continued aerobic promotion, may be essential to supporting balanced physical development and reducing long-term cardiometabolic and musculoskeletal risk. These findings suggest that a national physical fitness surveillance system should be based on a concise battery of low-cost and easily administered field tests once per year. These tests should include the 20-meter shuttle run for CRF, the SBJ, the medicine ball throw and the handgrip strength test for lower- and upper-limb muscular strength; the 10 × 5-meter shuttle run for speed-agility; and a static balance test. In the present study, this battery of tests captured distinct age-specific patterns of improvement, stagnation or decline across fitness domains. Additionally, including simple motor competence assessments (i.e. KTK test^39^ or the MOBAK test^42^) may be beneficial, as motor competence is strongly associated with physical activity participation^43^ and can help identify early deficits in movement proficiency that fitness measures alone do not capture.

### Limitations of this study

It is important to acknowledge several limitations of this study. Firstly, while we categorized participants into age groups for our analysis, we did not gather data on other potential confounding factors that could influence weight-related PF disparities, such as socioeconomic status. However, we have adjusted the models for maturity offset to account for differences in maturation. Although the study was analyzed as repeated cross-sectional, the lack of individual identifiers prevented formal longitudinal tracking; however, sensitivity analyses using mixed-effects models suggested that potential within-child clustering did not affect the results. Additionally, we did not collect any clinical outcomes, such as systolic and diastolic blood pressure, which could have strengthened our findings related to health. Lastly, we are unable to determine the causes behind the observed changes in PF, as we lack data on possible mediators like concurrent changes in diet, sleep, sedentary behavior, or psychological factors related to the pandemic (e.g. stress, anxiety, or depression).

## Conclusions

This study demonstrates the temporal trends in PF of primary school children in Northern Italy. The notable improvements in CRF, balance, speed-agility and lower limb MS underscore a change in PF trends among Italian children. However, no improvements were noted in upper-limb MS, highlighting the need for targeted interventions for this domain. For these reasons, we recommend implementing a nationwide surveillance system to monitor PF levels in children, to accurate evaluate the PF levels. Moreover, a future national system and future studies should include clinical data (i.e. systolic and diastolic blood pressure and waist circumference) providing a comprehensive and cost-effective assessment of children’s health. Future studies are needed to understand long-term trends in children’s PF and to confirm our results. A nationwide PF surveillance system would enable the continuous tracking of fitness trends, helping policymakers identify periods of decline and develop timely interventions.

## Supplementary Material

supplementary_files_fdag020

## Data Availability

Due to privacy concerns, the datasets used in this study are not publicly available. However, researchers can request access to specific individual-level data for academic use only, within 36 months following the publication date, after de-identification. Proposals should be directed to the corresponding author. Upon proposal acceptance, requestors will be granted access to the data after signing a data access agreement.

## Notes

**Alessandro Gatti,** Dr.

**Matteo Giuriato,** Dr.

**Matteo Vandoni,** Prof.

**Giovanni Messina,** Prof.

**Rita Malavolta,** Dr.

**Caterina Cavallo,** Dr.

**Agnese Pirazzi,** Dr.

**Vittoria Carnevale Pellino,** Dr.

**Nicola Lovecchio,** Prof.

**Stefano Lazzer,** Prof.
