# Supplementary material for: Temporal trends of physical fitness in northern Italian children (2014–2019): a repeated cross-sectional study
Source: J Public Health (Oxf). 2026 Mar 5;48(2):399–410. doi: 10.1093/pubmed/fdag020 (PMC13223575; doi:10.1093/pubmed/fdag020)
Supplement: supplementary_files_fdag020 [file supplementary_files_fdag020.zip › Figure S12_fdag020.docx]

**
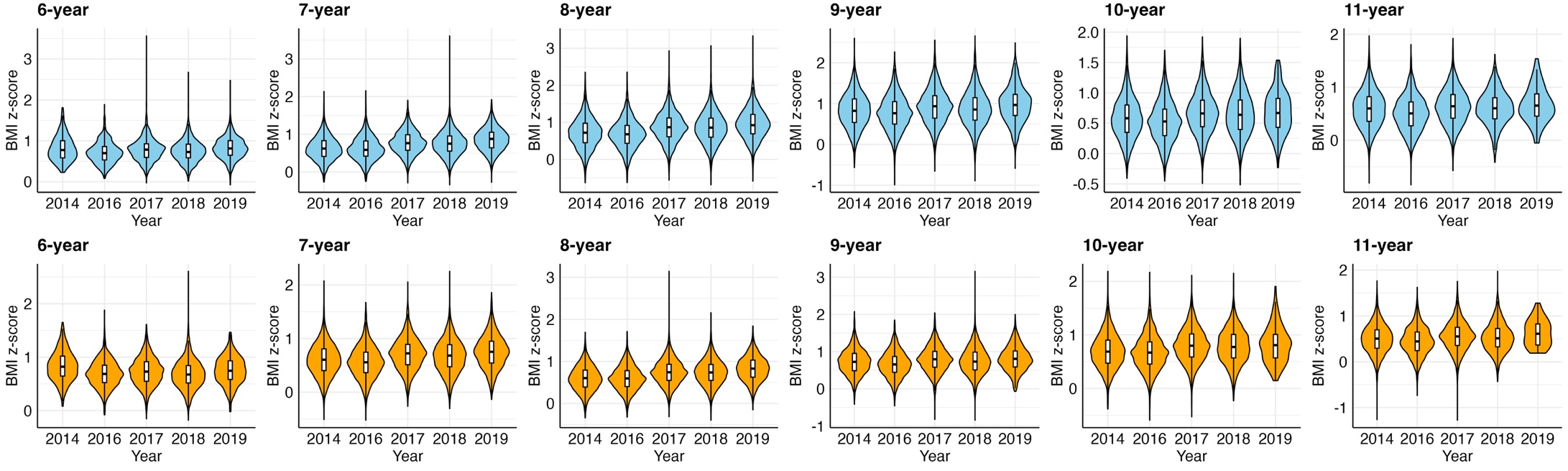
**

**Figure S12.** Temporal trends in BMI z-score for 6 to 11 years old girls (in blue) and for boys (in orange) from 2014 to 2019. The values in the graph are estimates obtained in the regression, adjusted for BMI z-score and Peak Height Velocity.
