# Supplementary material for: Temporal trends of physical fitness in northern Italian children (2014–2019): a repeated cross-sectional study
Source: J Public Health (Oxf). 2026 Mar 5;48(2):399–410. doi: 10.1093/pubmed/fdag020 (PMC13223575; doi:10.1093/pubmed/fdag020)
Supplement: supplementary_files_fdag020 [file supplementary_files_fdag020.zip › Figure S3_fdag020.docx]

**
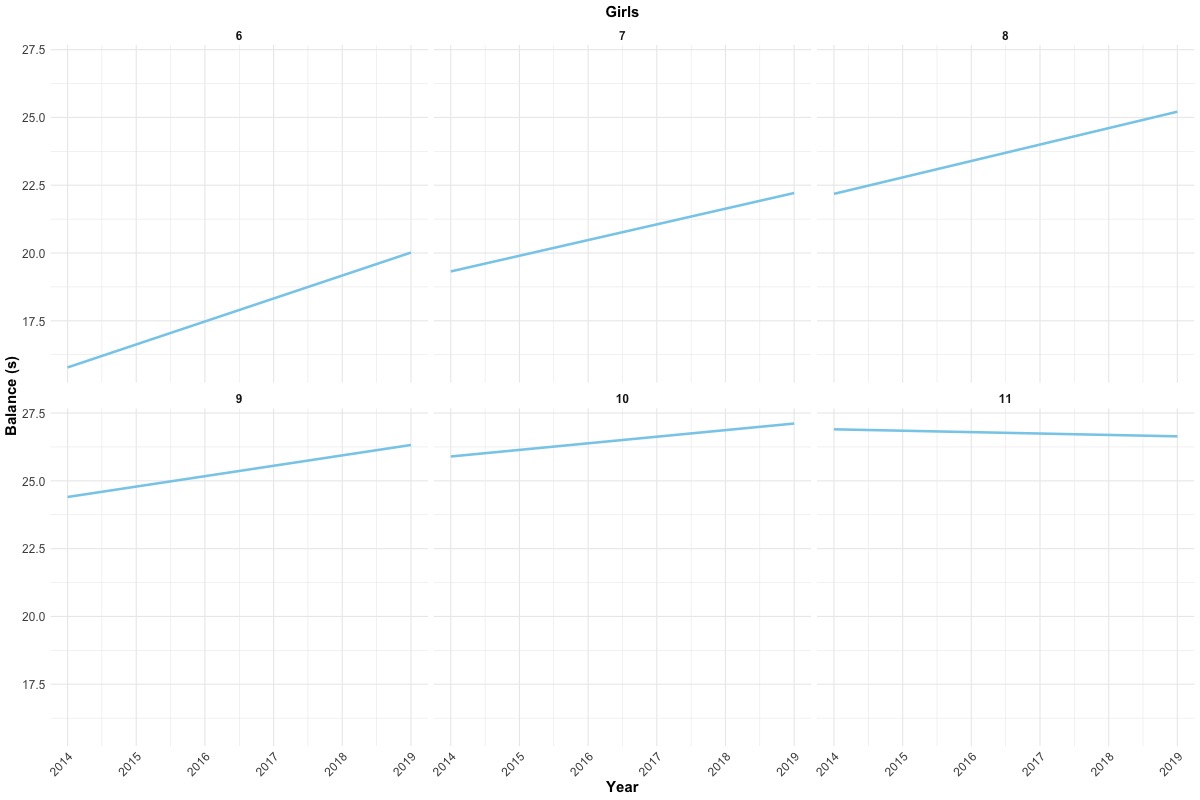
**

**Figure S2.** Temporal trends in balance (seconds) domains for 6 (upper left corner) to 11 (lower right corner) years old girls from 2014 to 2019. The values in the graph are estimates obtained in the generalized linear mixed model, adjusted for BMI z-score and Peak Height Velocity.
