# Supplementary material for: Temporal trends of physical fitness in northern Italian children (2014–2019): a repeated cross-sectional study
Source: J Public Health (Oxf). 2026 Mar 5;48(2):399–410. doi: 10.1093/pubmed/fdag020 (PMC13223575; doi:10.1093/pubmed/fdag020)
Supplement: supplementary_files_fdag020 [file supplementary_files_fdag020.zip › Table S1_fdag020.docx]

**Table S1.** Generalized Linear Mixed Model results showing the association between balance time (seconds) and year, grouped by age

| Age group | Boys | | | Girls | | |
| --- | --- | --- | --- | --- | --- | --- |
|  | b | R^2^ | *p*-value | b | R^2^ | *p*-value |
| 6 | 0.65 (0.41, 0.88) | 0.27 | < 0.001 | 0.93 (0.68, 1.17) | 0.33 | < 0.001 |
| 7 | 0.03 (-0.06, 0.13) | 0.27 | 0.494 | 0.27 (0.19, 0.36) | 0.30 | < 0.001 |
| 8 | 0.09 (0.00, 0.18) | 0.26 | 0.049 | 0.21 (0.13, 0.29) | 0.35 | < 0.001 |
| 9 | -0.14 (-0.23, -0.06) | 0.36 | 0.001 | -0.04 (-0.12, 0.04) | 0.43 | 0.300 |
| 10 | -0.34 (-0.43, -0.25) | 0.41 | < 0.001 | -0.23 (-0.31, -0.16) | 0.49 | < 0.001 |
| 11 | -0.52 (-0.60, -0.44) | 0.54 | < 0.001 | -0.45 (-0.52, -0.39) | 0.58 | < 0.001 |

*Notes: The coefficients (b) are reported as unstandardized with the 95% confidence interval.*
