# Supplementary material for: Temporal trends of physical fitness in northern Italian children (2014–2019): a repeated cross-sectional study
Source: J Public Health (Oxf). 2026 Mar 5;48(2):399–410. doi: 10.1093/pubmed/fdag020 (PMC13223575; doi:10.1093/pubmed/fdag020)
Supplement: supplementary_files_fdag020 [file supplementary_files_fdag020.zip › Table S2_fdag020.docx]

**Table S2.** Generalized Linear Mixed Model results showing the association between VO_2_ max (ml/kg/min) and year, grouped by age

| Age group | Boys | | | Girls | | |
| --- | --- | --- | --- | --- | --- | --- |
|  | b | R^2^ | *p*-value | b | R^2^ | *p*-value |
| 6 | 0.32 (0.08, 0.55) | 0.55 | 0.008 | 0.43 (0.24, 0.62) | 0.64 | < 0.001 |
| 7 | 0.53 (0.34, 0.71) | 0.49 | < 0.001 | 0.45 (0.27, 0.62) | 0.44 | < 0.001 |
| 8 | 0.54 (0.36, 0.73) | 0.42 | < 0.001 | 0.58 (0.42, 0.75) | 0.49 | < 0.001 |
| 9 | 0.74 (0.53, 0.96) | 0.46 | < 0.001 | 0.89 (0.70, 1.09) | 0.46 | < 0.001 |
| 10 | 1.06 (0.76, 1.36) | 0.53 | < 0.001 | 1.16 (0.89, 1.43) | 0.63 | < 0.001 |
| 11 | 1.29 (0.89, 1.69) | 0.68 | < 0.001 | 1.26 (0.86, 1.66) | 0.65 | < 0.001 |

*Notes: The coefficients (b) are reported as unstandardized with the 95% confidence interval.*
