# Supplementary material for: Temporal trends of physical fitness in northern Italian children (2014–2019): a repeated cross-sectional study
Source: J Public Health (Oxf). 2026 Mar 5;48(2):399–410. doi: 10.1093/pubmed/fdag020 (PMC13223575; doi:10.1093/pubmed/fdag020)
Supplement: supplementary_files_fdag020 [file supplementary_files_fdag020.zip › Table S3_fdag020.docx]

**Table S3.** Generalized Linear Mixed Model results showing the association between medicine ball throw (cm) and year, grouped by age

| Age group | Boys | | | Girls | | |
| --- | --- | --- | --- | --- | --- | --- |
|  | b | R^2^ | *p*-value | b | R^2^ | *p*-value |
| 6 | 3.69 (2.44, 4.93) | 0.44 | < 0.001 | 3.40 (2.26, 4.54) | 0.47 | < 0.001 |
| 7 | 3.70 (3.14, 4.26) | 0.58 | < 0.001 | 3.04 (2.55, 3.52) | 0.61 | < 0.001 |
| 8 | 2.82 (2.22, 3.42) | 0.58 | < 0.001 | 2.75 (2.22, 3.28) | 0.63 | < 0.001 |
| 9 | 1.23 (0.57, 1.89) | 0.64 | < 0.001 | 1.41 (0.81, 2.02) | 0.66 | < 0.001 |
| 10 | 1.02 (0.31, 1.74) | 0.70 | 0.005 | 1.42 (0.77, 2.08) | 0.75 | < 0.001 |
| 11 | 0.40 (-0.29, 1.10) | 0.80 | 0.259 | 0.52 (-0.12, 1.16) | 0.83 | 0.109 |

*Notes: The coefficients (b) are reported as unstandardized with the 95% confidence interval.*
