# Supplementary material for: Temporal trends of physical fitness in northern Italian children (2014–2019): a repeated cross-sectional study
Source: J Public Health (Oxf). 2026 Mar 5;48(2):399–410. doi: 10.1093/pubmed/fdag020 (PMC13223575; doi:10.1093/pubmed/fdag020)
Supplement: supplementary_files_fdag020 [file supplementary_files_fdag020.zip › Table S4_fdag020.docx]

**Table S4.** Generalized Linear Mixed Model results showing the association between standing broad jump (cm) and year, grouped by age

| Age group | Boys | | | Girls | | |
| --- | --- | --- | --- | --- | --- | --- |
|  | b | R^2^ | *p*-value | b | R^2^ | *p*-value |
| 6 | 0.45 (0.00, 0.90) | 0.40 | 0.051 | 0.59 (0.17, 1.01) | 0.43 | 0.006 |
| 7 | 0.88 (0.69, 1.07) | 0.55 | < 0.001 | 0.93 (0.76, 1.10) | 0.56 | < 0.001 |
| 8 | 0.90 (0.71, 1.10) | 0.55 | < 0.001 | 0.90 (0.72, 1.09) | 0.58 | < 0.001 |
| 9 | 0.51 (0.31, 0.71) | 0.59 | < 0.001 | 0.52 (0.32, 0.71) | 0.63 | < 0.001 |
| 10 | 0.50 (0.28, 0.73) | 0.67 | < 0.001 | 0.55 (0.35, 0.76) | 0.72 | < 0.001 |
| 11 | 0.17 (-0.02, 0.36) | 0.83 | 0.086 | 0.23 (0.04, 0.41) | 0.84 | 0.016 |

*Notes: The coefficients (b) are reported as unstandardized with the 95% confidence interval.*
