# Supplementary material for: Temporal trends of physical fitness in northern Italian children (2014–2019): a repeated cross-sectional study
Source: J Public Health (Oxf). 2026 Mar 5;48(2):399–410. doi: 10.1093/pubmed/fdag020 (PMC13223575; doi:10.1093/pubmed/fdag020)
Supplement: supplementary_files_fdag020 [file supplementary_files_fdag020.zip › Table S5_fdag020.docx]

**Table S5.** Generalized Linear Mixed Model results showing the association between shuttle run time (s) and year, grouped by age

| Age group | Boys | | | Girls | | |
| --- | --- | --- | --- | --- | --- | --- |
|  | b | R^2^ | *p*-value | b | R^2^ | *p*-value |
| 6 | -0.39 (-0.52, -0.26) | 0.45 | < 0.001 | -0.32 (-0.45, -0.19) | 0.45 | < 0.001 |
| 7 | -0.38 (-0.47, -0.30) | 0.35 | < 0.001 | -0.35 (-0.44, -0.27) | 0.40 | < 0.001 |
| 8 | -0.32 (-0.41, -0.23) | 0.35 | < 0.001 | -0.48 (-0.57, -0.39) | 0.41 | < 0.001 |
| 9 | -0.33 (-0.44, -0.21) | 0.36 | < 0.001 | -0.11 (-0.21, 0.00) | 0.43 | 0.045 |
| 10 | -0.11 (-0.23, 0.02) | 0.41 | 0.101 | -0.14 (-0.25, -0.02) | 0.48 | 0.023 |
| 11 | -0.16 (-0.31, -0.01) | 0.51 | 0.037 | -0.22 (-0.40, -0.05) | 0.44 | 0.012 |

*Notes: The coefficients (b) are reported as unstandardized with the 95% confidence interval.*
