# Supplementary figures and images for: Temporal trends of physical fitness in northern Italian children (2014–2019): a repeated cross-sectional study
Source: J Public Health (Oxf). 2026 Mar 5;48(2):399–410. doi: 10.1093/pubmed/fdag020 (PMC13223575; doi:10.1093/pubmed/fdag020)

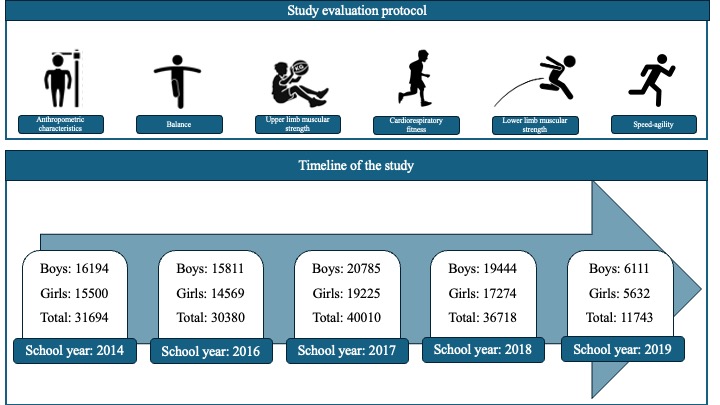

Supplement: supplementary_files_fdag020 [file supplementary_files_fdag020.zip › Figure_S1_fdag020.jpeg]
